# Association between Depressive Symptom Trajectory and Chronic Kidney Disease Progression: Findings from the Chronic Renal Insufficiency Cohort Study

**DOI:** 10.34067/KID.0000000000000087

**Published:** 2023-02-23

**Authors:** Celestin Missikpode, Ana C. Ricardo, Julia Brown, Ramon A. Durazo-Arvizi, Michael J. Fischer, Rosalba Hernandez, Anna C. Porter, Judith A. Cook, Amanda Anderson, Jacquie Dolata, Harold I. Feldman, Edward Horwitz, Claudia Lora, Julie Wright Nunes, Panduranga S. Rao, James P Lash

**Affiliations:** 1 Department of Medicine, University of Illinois at Chicago, Chicago, Illinois; 2 Keck School of Medicine, University of Southern California, Los Angeles, California; 3 Medical Service, Jesse Brown VA Medical Center, Chicago, Illinois; 4 School of Social Work, University of Illinois at Urbana-Champaign, Urbana, Illinois; 5 Department of Psychiatry, University of Illinois at Chicago, Chicago, Illinois; 6 Department of Epidemiology, Tulane University School of Public Health and Tropical Medicine, New Orleans, Louisiana; 7 Division of Nephrology and Hypertension, MetroHealth Medical Center, Case Western Reserve University, Cleveland, Ohio; 8 Center for Clinical Epidemiology and Biostatistics, Perelman School of Medicine at the University of Pennsylvania, Philadelphia, Pennsylvania; 9 Department of Biostatistics, Epidemiology, and Informatics, Perelman School of Medicine at the University of Pennsylvania, Philadelphia, Pennsylvania; 10 Department of Medicine, University of Michigan, Ann Arbor, Michigan

**Keywords:** chronic kidney disease, depression, end-stage kidney disease, progression of chronic renal failure, renal progression

## Abstract

**Key Points:**

- Depressive symptoms are largely stable over time among individuals with mild-to-moderate CKD
- Low educational attainment, cigarette smoking, and poor quality of life are associated with persistent depressive symptoms
- Persistent depressive symptoms are associated with nonlinear and rapid decline in kidney function

**Background:**

Although depression is highly prevalent among individuals with CKD, little is known about the course of depressive symptoms over time. We characterized trajectories of depressive symptoms and CKD progression and evaluated the association between depressive symptoms trajectory and CKD progression.

**Methods:**

Two thousand three hundred sixty-one individuals with mild-to-moderate CKD enrolled in the Chronic Renal Insufficiency Cohort Study were analyzed. The Beck Depression Inventory (BDI) was used to assess depressive symptoms at baseline and biennially. Higher BDI scores indicate worse depressive symptoms. eGFR was calculated using the 2021 CKD-EPI equation. Group-based trajectory models were used to determine trajectories of BDI score and eGFR change over time. Multinomial logistic regression was used to examine factors associated with BDI trajectories and to evaluate the association of BDI trajectories with eGFR change.

**Results:**

Over 8 years of follow-up, three patterns of depressive symptoms were identified: persistently low BDI score (57.7%), persistently moderate BDI score (33.1%), and persistently high BDI score (9.2%). Three eGFR trajectory groups were identified: nonlinear, rapid eGFR decline (21.5%); linear, expected eGFR decline (54.8%); and stable eGFR (23.7%). Predictors of persistently moderate and high BDI trajectories included low educational attainment, smoking, and poor quality of life. Compared with those with a persistently low BDI score, the odds for nonlinear, rapid eGFR decline were higher for those with persistently moderate BDI scores (odds ratio [OR], 1.45; 95% confidence interval [CI], 1.04 to 2.03) and persistently high BDI scores (OR, 1.90; 95% CI, 1.02 to 3.56). No association between moderate BDI score and linear, expected eGFR decline was observed.

**Conclusions:**

Depressive symptoms remained largely stable among individuals with mild-to-moderate CKD, and persistently moderate and high BDI scores were associated with nonlinear, rapid eGFR decline. Future work is needed to better understand the interplay between depression and CKD progression.

## Graphical abstract

**Figure:**
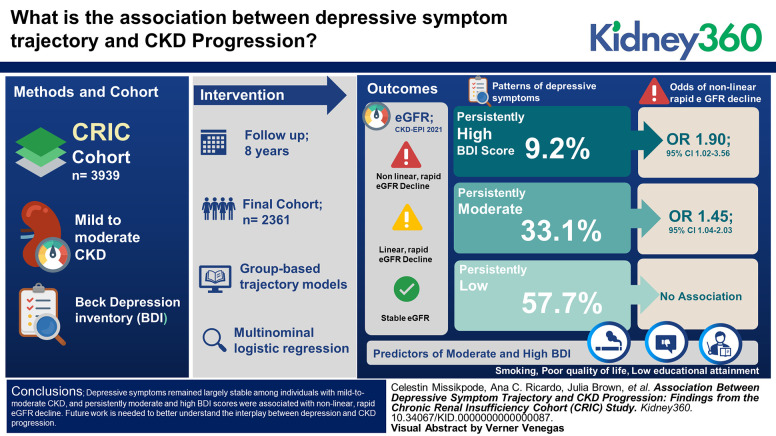

## Introduction

Depression is a significantly debilitating mental disorder and has been recognized as a growing burden to global public health.^1,2^ Symptoms of depression can vary, ranging from decreased socioemotional well-being^3^ to physical impairment^2^ and lower productivity in the workplace.^4^ Different longitudinal patterns of depressive symptoms have been reported in the general population where some individuals remain stable with few symptoms, while others experience decreasing, increasing, or consistently high symptoms over time.^5^ Among individuals with CKD, depression is highly prevalent ranging from 15% to >50%.^6–8^ Although evidence suggests that depression is very common among individuals with CKD, little is known about the course and heterogeneity of depressive symptoms over time in this high-risk population. A greater understanding of the course of depressive symptoms over time and characteristics that describe this course may inform intervention and prevention strategies.

Studies have shown an association between depressive symptoms and CKD progression.^9,10^ However, CKD progression was defined as the development of ESKD or a prespecified reduction in kidney function. This approach may hinder our ability to gain insights into the association of depressive symptoms with trajectories of CKD progression. For example, CKD progression demonstrates individual variability over time where some individuals experience prolonged periods of nonprogression and others a linear or nonlinear rapid decline in kidney function.^11^

The objectives of this study were to characterize the longitudinal trajectories of depressive symptoms and eGFR among individuals with mild-to-moderate CKD, evaluate predictors of depressive symptoms trajectory, and examine the association of depressive symptoms trajectory with CKD progression.

## Methods

### Study Population

**Figure 1:**
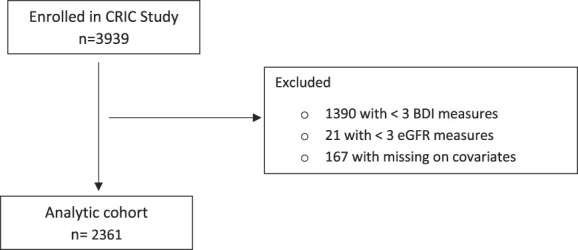
Flowchart of analytical sample selection.

Data from the Chronic Renal Insufficiency Cohort (CRIC) Study were analyzed. Details of the design and protocol of CRIC are published elsewhere.^12^ In brief, the CRIC Study is a multicenter, prospective cohort study that was conducted at seven US clinical centers and enrolled 3939 individuals aged 21–75 years at baseline with an eGFR between 20 and 70 ml/min per 1.73 m^2^. Institutional review boards at each participating center approved this study in accordance with the Declaration of Helsinki, and written informed consent was obtained from all participants. For this study, we included 2361 participants with at least three time points of data available for eGFR and the Beck Depression Inventory (BDI) during the study period (Figure 1). Participants were followed until death, withdrawal from this study, or December 2019 when the database was locked for analysis.

### Exposures

At baseline and every two years, BDI was used to assess depressive symptoms. The BDI is a 21-item self-report instrument which is a validated and reliable tool for screening for the existence and severity of depressive symptoms.^13–19^ Each item is scored using a 4-point Likert scale, with derivation of a composite score ranging from 0 to 63 calculated by summing individual survey items. A BDI score of 11 or higher has been considered to be a clinically meaningful level of depressive symptoms in patients with CKD.^16^ A data-driven approach was used to systematically separate individuals into latent groups of similar BDI trajectories.

### Outcome Measures

GFR was estimated using the 2021 CKD-EPI equation that includes serum creatinine level, age, and sex.^20^ Serum creatinine was measured on a Roche Modular P Chemistry Analyzer using a creatinase enzymatic method (Roche Diagnostics, Indianapolis, IN). GFR was estimated at baseline and every annual follow-up visit. For participants who developed ESKD or died during the study period, eGFR before the onset of ESKD or death was used. ESKD was defined as initiation of dialysis therapy or kidney transplantation, with ascertainment performed through semiannual surveillance by study personnel and further confirmed by cross-linkage with the US Renal Data System. For each participant, change in eGFR at each annual follow-up visit was defined as the difference between the baseline eGFR and the follow-up eGFR.

### Covariates

Covariates examined in this study were sociodemographic characteristics (age, sex, race and ethnicity, and educational attainment), lifestyle behaviors, medical history, and medication use. Race and ethnicity was categorized as non-Hispanic White, non-Hispanic Black, and Hispanic. Current cigarette smoking was determined by self-report. Physical activity was assessed as metabolic equivalent of task (MET), and participants were categorized as MET score ≥6 (yes versus no).^21^ Weight and height were measured using standard protocols, and body max index (BMI) was calculated as weight in kilograms divided by height in meters squared. Hypertension was defined as mean systolic blood pressure 140 mm Hg or greater, mean diastolic blood pressure 90 mm Hg or greater, or use of antihypertensive medications. The history of cardiovascular disease (*e.g.*, chronic heart failure, myocardial infarction, or coronary revascularization) was self-reported. Diabetes mellitus was defined as a fasting glucose level ≥126 mg/dl, a nonfasting glucose level ≥200 mg/dl, or use of antihyperglycemic medications. Additional assay measures included 24-hour urine total protein and hemoglobin. Anemia was defined as hemoglobin <13 g/dl for men and <12 g/dl for women. Documentation of current medication use included ascertainment of antidepressants and angiotensin-converting enzyme inhibitor/angiotensin receptor blocker (ACEI/ARB). Information on quality of life was collected using the Kidney Disease Quality of Life (KDQOL-36) questionnaire, which is specifically tailored for patients with kidney disease.^22,23^

### Statistical Methods

Descriptive statistics are summarized as means (SD) or medians (IQR) for continuous variables and frequencies (proportions) for categorical variables. The chi-squared test was used to compare categorical variables and *t* test or Wilcoxon rank-sum test to compare continuous variables. Group-based trajectory analysis was used to identify qualitatively distinct CKD subpopulations with similar BDI scores over time.^24–26^ Similar methodology was used to classify participants with CKD according to trajectory of change in eGFR over time adjusting for baseline eGFR. Participants were included in our analytic cohort if they had at least three measurements on BDI scores or eGFR. For those with at least three measurements but incomplete follow-up data on BDI or eGFR, only their available data were used in group-based trajectory models. The group-based trajectory approach was designed to identify clusters of individuals following a similar pattern over time on a given variable (in this case, BDI score and change in eGFR). Time series data for BDI scores and change in eGFR were modeled assuming a censored normal distribution.^25^ The censored normal distribution is designed for the analysis of repeatedly measured (approximately) continuous scales and can accommodate the possibility of clustering at the scale minimum, maximum, or both.^25^ Different models with a varying number of groups and shapes were compared to find the models that best described BDI score and change in eGFR. The analytical framework allows estimation of up to a fourth-order polynomial. We began with a two-group model fitted using a quartic degree polynomial function, and then, we increased the number of groups up to 5. After we identified the optimal number of groups, we reduced the polynomial orders until the highest order polynomial for each group was significant at the level of *α*=0.05. The optimal model was determined based on Bayesian information criterion, Bayes factors, meaningfulness of group proportion, and trajectory shapes.^26^

Multinomial logistic regression model was used to predict BDI score group membership. The relationship between trajectories of BDI scores and patterns of change in eGFR was examined using multinomial logistic regression, adjusting for covariates. Analyses were conducted using SAS version 9.4 (SAS Institute, Cary, NC), and graphs were produced in R.

## Results

At study entry, participants were on average age 58 (SD, 10.3) years, 47% were female, 49% self-identified as non-Hispanic White, 38% as non-Hispanic Black, and 13% as Hispanic. Compared with participants excluded at baseline (*n*=1578), those included (*n*=2361) were slightly older (58 versus 57 years), more likely to be female (47% versus 42%), non-Hispanic White (49% versus 30.5%), have higher eGFR (45.6 versus 37.1 ml/min per 1.73 m^2^), have lower BDI scores (7.1 versus 9.5), and were less likely to have diabetes (42.1% versus 57.9%), hypertension (83.7% versus 89.7%), and cardiovascular disease (29.7% versus 39%) (Supplemental Table 1).

During a median (IQR) follow-up time of 8 (6–8) years, the median (range) number of BDI measurements was 4 (3–5), and the median (range) number of eGFR measurements was 9 (3–9). The mean (SD) BDI score was 7.1 (7.1) at baseline.

**Figure 2:**
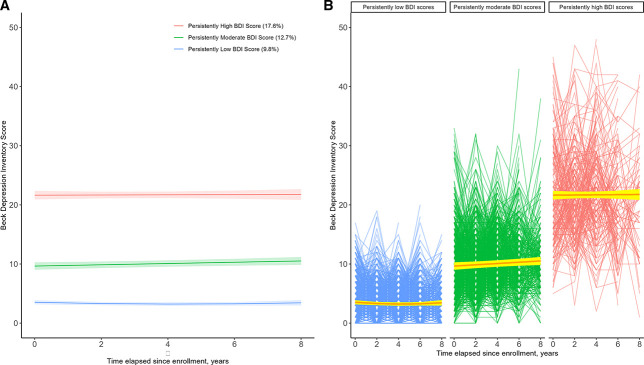
**Beck Depression Inventory (BDI) Score.** (A) Mean trajectories and 95% confidence intervals of BDI Score by time elapsed since enrollment. (B) Individual patterns of BDI Score over time by trajectory group membership and their estimated mean lines.

Figure 2A shows three mean BDI trajectories estimated by the selected group-based trajectory model, plotted by time elapsed since enrollment. Patterns of BDI scores among participants demonstrated a high degree of stability over time as follows: persistently low BDI score in 1363 (57.7%), persistently moderate BDI in 782 (33.1%), and persistently high BDI score in 216 (9.2%). The average posterior probabilities for all three BDI trajectory groups were 0.93, 0.88, and 0.95, far greater than the recommended threshold of 0.7.^26^ This indicates that the model assigned participants to different BDI groups with little ambiguity. Within each BDI trajectory group membership, participants exhibited little to no change in BDI score over time (Figure 2B).

**Figure 3:**
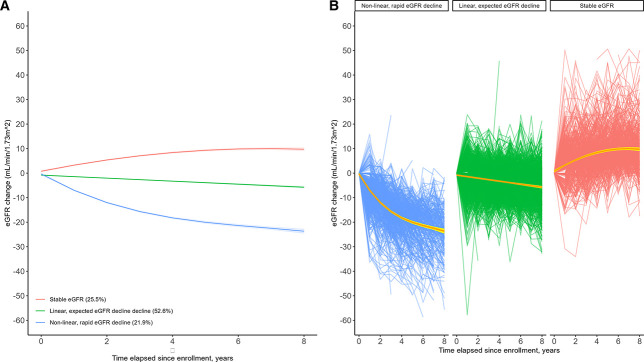
**Change in eGFR by time elapsed since enrollment.** (A) Mean trajectories and 95% confidence intervals of eGFR change (B) Individual patterns of eGFR change.

A three-class model was found to provide the best characterization of patterns of eGFR change over time (Figure 3A). The three groups identified were as follows: nonlinear, rapid eGFR decline in 507 (21.5%); linear, expected eGFR decline in 1293 (54.8%); and stable eGFR in 561 (23.7%). The posterior probabilities for the three groups were satisfactory (*i.e.*, 0.95, 0.94, and 0.94 for nonlinear, rapid eGFR decline; linear, expected eGFR decline; and stable eGFR, respectively), and uncertainty on group assignment was not a factor. Within each eGFR trajectory group membership, participants largely followed the group average patterns (Figure 3B).

Compared with individuals with persistently low BDI score, those with persistently high BDI score were slightly younger (55.5 years versus 58.6 years), more likely to be female (61.6% versus 42.6%), and non-Hispanic Black or Hispanic (Table 1). In addition, participants with persistently high versus low BDI scores were more likely to have less than a high school education (26.9% versus 10.3%) and lack health insurance (12.0% versus 4.1%). Individuals with persistently high BDI score were more likely to be smokers (24.5%) compared with those with persistently low BDI score (7.0%). Compared with participants with persistently low BDI score, those with high BDI score were less likely to be physically active (13.9% versus 32.4%) and alcohol drinkers (56.9% versus 69.5%). Furthermore, lower quality of life was more common among participants in the moderate and high BDI trajectory groups. In addition, those with a persistently high BDI score were more likely to have diabetes, prevalent cardiovascular disease, and BMI ≥30 kg/m^2^. The use of antidepressants was low across all groups but higher for those with persistently moderate and high BDI score.

**Table 1:** Baseline characteristics by trajectory of Beck Depression Inventory Score

Variables	Persistently Low Beck Depression Inventory Score (*n*=1363)	Persistently Moderate Beck Depression Inventory Score (*n*=782)	Persistently High Beck Depression Inventory Score (*n*=216)	*P* Value
Age, yr, mean (SD)	58.6 (10.4)	58.5 (10.2)	55.5 (9.4)	0.0001
Age <50 yr, %	239 (17.5)	147 (18.8)	55 (25.5)	0.02
Sex, female, %	580 (42.6)	396 (50.6)	133 (61.6)	<0.0001
Race, %				<0.0001
Non-Hispanic White	739 (54.2)	341 (43.6)	76 (35.2)	
Non-Hispanic Black	473 (34.7)	326 (41.7)	101 (46.8)	
Hispanic	151 (11.1)	115 (14.7)	39 (18.1)	
Less than high school, %	140 (10.3)	160 (20.5)	58 (26.9)	<0.0001
No health insurance, %	56 (4.1)	61 (7.8)	26 (12.0)	<0.0001
Current smoker, %	95 (7.0)	95 (12.2)	53 (24.5)	<0.0001
Alcohol use, %	947 (69.5)	501 (64.1)	123 (56.9)	0.0003
Any activities with MET score ≥6, %	441 (32.4)	156 (20.0)	30 (13.9)	<0.0001
Diabetes, %	512 (37.6)	372 (47.6)	111 (51.4)	<0.0001
Systolic BP, mean (SD), mmHg	123.8 (19.2)	125.5 (20.4)	125.9 (19.6)	0.08
Diastolic BP, mean (SD), mmHg	70.8 (12.0)	69.8 (12.2)	71.4 (11.1)	0.12
Cardiovascular disease, %	330 (24.2)	278 (35.6)	92 (42.6)	<0.0001
BMI ≥30 kg/m^2^, %	697 (51.1)	473 (60.5)	142 (65.7)	<0.0001
Quality of life—physical composite, mean (SD)	46.3 (10.1)	39.0 (11.5)	33.7 (9.9)	<0.0001
Quality of life—mental composite, mean (SD)	55.3 (6.66)	47.6 (10.1)	36.2 (9.6)	<0.0001
eGFR, mean (SD), ml/min per 1.73 m^2^	46.7 (14.33)	44.3 (15.2)	43.3 (15.3)	<0.0001
Hemoglobin, mean (SD), mg/dl	13.0 (1.7)	12.7 (1.6)	12.4 (1.6)	<0.0001
24H urine protein, median, g/24H	0.11	0.13	0.19	<0.0001
High sensitivity CRP, mean (SD), mg/L	4.6 (8.0)	5.7 (10.1)	6.2 (10.3)	0.003
ACEI/ARB use, %	934 (68.5)	552 (70.6)	130 (60.2)	0.01
Antidepressant use, %	149 (10.9)	198 (25.3)	95 (44.0)	<0.0001

BMI, body max index; MET, metabolic equivalent of task; CRP, C-reactive protein; ACEI/ARB, angiotensin-converting enzyme inhibitor/angiotensin receptor blocker.

### Multivariable Predictors of BDI Trajectory Assignment

In multivariable analyses, individuals with less than a high school education were more likely to have a persistently moderate BDI score (odds ratio [OR], 1.46; 95% confidence interval [CI], 1.06 to 2.02) and to have a persistently high BDI score (OR, 1.54; 95% CI, 0.91 to 2.61) (Table 2). Current smokers were more likely to have a persistently moderate BDI score (OR, 1.41; 95% CI, 0.98 to 2.03) and more likely to have a persistently high BDI score (OR, 2.97; 95% CI, 1.70 to 5.18). Higher HRQOL-36 physical and mental composites were associated with lower odds of membership in the moderate and high BDI trajectory groups. The use of antidepressants was also significantly associated with membership in persistently moderate and high BDI trajectories.

**Table 2:** Factors associated with Beck Depression Inventory trajectory

Baseline Variables	Odds Ratio (95% CI)	
	Persistently Moderate Beck Depression Inventory Score	Persistently High Beck Depression Inventory Score
Less than high school	1.46 (1.06 to 2.02)	1.54 (0.91 to 2.61)
Current smoking	1.41 (0.98 to 2.03)	2.97 (1.70 to 5.18)
Quality of life—physical composite per one-unit increase	0.94 (0.93 to 0.95)	0.89 (0.87 to 0.91)
Quality of life—mental composite per one-unit increase	0.90 (0.88 to 0.91)	0.80 (0.78 to 0.82)
Antidepressant use	1.66 (1.25 to 2.21)	2.94 (1.89 to 4.57)

Referent group: Persistently low BDI score. Adjusted for clinical center, age, sex, race, educational attainment, health insurance status, smoking status, alcohol use, physical activity, diabetes, hypertension, cardiovascular disease, anemia, obesity, quality of life, eGFR, 24-hour urine protein, high-sensitivity CRP, antidepressant use, and ACEI/ARB use. CI, confidence interval; BDI, Beck Depression Inventory.

### Association of BDI Trajectory with eGFR Change over Time

**Figure 4:**
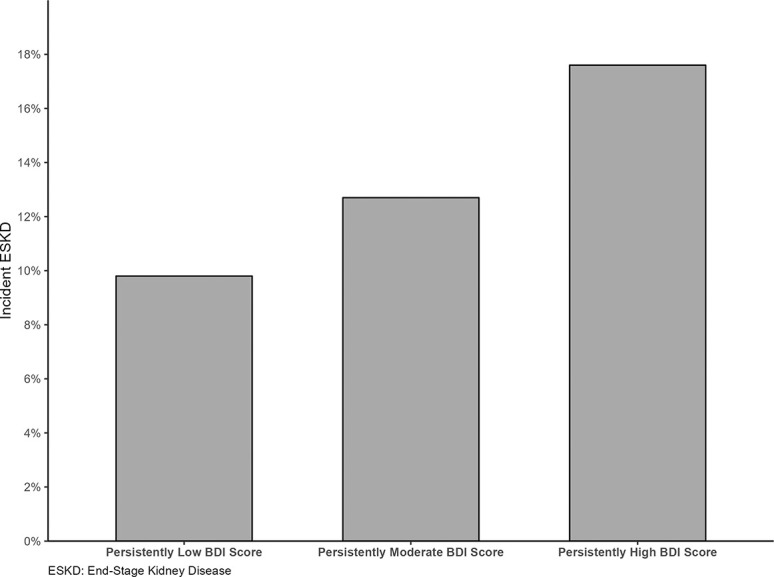
Incidence proportions of end-stage renal disease across trajectory groups of Beck Depression Inventory (BDI) Score.

Over a median follow-up of 8 years, 270 (11.4%) participants developed ESKD and 238 (10.1%) died. A significantly higher proportion of individuals with persistently high BDI scores developed ESKD during the study period (17.6%), compared with those with persistently moderate and low BDI scores (12.7% and 9.8%, respectively) (*P*=0.0015) (Figure 4). A significant higher proportion of individuals with nonlinear, rapid eGFR decline developed ESKD (31.4%) compared with those with linear, expected eGFR decline and stable eGFR (8.4% and 0.4%, respectively) (*P*<0.0001). There was a significant association of BDI trajectory with patterns of eGFR change (Table 3). Compared with those with a persistently low BDI score, the adjusted odds of nonlinear, rapid eGFR decline were higher for individuals with persistently high BDI scores (OR, 1.90; 95% CI, 1.02 to 3.56) and with persistently moderate BDI score (OR, 1.45; 95% CI, 1.04 to 2.03) (Table 3). Persistently high BDI scores were also associated with linear, expected eGFR decline (OR, 1.79; 95% CI, 1.10 to 2.92). Persistently moderate BDI scores were not associated with linear, expected eGFR decline.

**Table 3:** Association between trajectories of Beck Depression Inventory Score and eGFR

BDI Trajectory Group	Odds Ratio (95% CI)	
	Linear, Expected eGFR Decline	Nonlinear, Rapid eGFR Decline
Persistently low BDI score	1.00	1.00
Persistently moderate BDI score	1.10 (0.85 to 1.42)	**1.45 (1.04 to 2.03)**
Persistently high BDI score	**1.79 (1.10 to 2.92)**	**1.90 (1.02 to 3.56)**

Referent group: Stable eGFR trajectory. Adjusted for clinical center, age, sex, race, educational attainment, health insurance status, smoking status, alcohol use, physical activity, diabetes, hypertension, cardiovascular disease, anemia, obesity, quality of life, eGFR, 24-hour urine protein, high-sensitivity CRP, antidepressant use, and ACEI/ARB use. CI, confidence interval.

## Discussion

We identified three trajectories of depressive symptoms on the basis of longitudinal BDI scores in adults with mild-to-moderate CKD followed up for eight years: persistently low, persistently moderate, and persistently high depressive symptoms. Sociodemographic characteristics (*i.e.*, lower educational attainment), lifestyle factors (*i.e.*, cigarette smoking), and quality of life were significant predictors of persistently moderate and high depressive symptoms. Multivariable longitudinal analyses revealed that persistently high and moderate levels of depressive symptoms were each independently associated with nonlinear, rapid eGFR decline.

Although previous studies have reported an association between depressive symptoms and CKD progression,^9,10^ they focused on the relationship of a single measurement of depressive symptoms with subsequent CKD progression. Our study reinforces and extends these findings by demonstrating an association between trajectories of depressive symptoms and patterns of eGFR progression. We found a graded increase in odds for rapid CKD progression among individuals with persistently moderate and persistently high BDI scores. We were unable to elucidate the temporal relationship between the two trajectory outcomes and therefore could not determine whether patterns of eGFR change were the cause or the result of depressive symptom trajectories. It is noteworthy that a significantly higher proportion of participants who were assigned to the persistently high BDI trajectory group developed ESKD during the study period. This finding raises the question of whether persistent depressive symptoms may have played a role in the decline of eGFR over time. However, the association between depressive symptoms and CKD progression is theorized to be bidirectional.^6^ On the one hand, depressive symptoms may have a direct effect on CKD progression due to factors which may include inflammation, treatment nonadherence, and unhealthy lifestyle.^27–29^ For example, the association between depressive symptoms and low medication adherence was reported in cross-sectional and longitudinal studies.^27^ On the other hand, individuals with CKD may develop depressive symptoms especially as they progress to ESKD. It has been suggested that negative illness perception itself can be associated with adverse mental health outcomes among individuals with CKD including anxiety and depression.^30,31^

Within each trajectory, participants exhibited little to no change in BDI score over time, highlighting the stability of depressive symptom levels among individuals with CKD. Persistence of depressive symptoms over extended periods of time has also been noted in cohorts with other chronic diseases, including chronic heart disease^32^ and HIV,^33^ and similar to our study has been found to be associated with adverse outcomes. In a study of 1700 HIV-positive women, chronic persistent depressive symptoms were associated with increased risk of death.^33^ In our cohort, a potential contributor to the persistence of depressive symptoms was the low use of antidepressant medications in this population. In fact, antidepressant medications were used by only 25.3% and 44.0% of participants in the persistently moderate and persistently high BDI score groups, respectively. Future studies are needed to evaluate whether treatment of depression influences CKD progression.

Our findings suggest that clinicians involved in the care of individuals with CKD should be vigilant to the presence of persistent depressive symptoms and arrange timely referral to mental health services. In addition, we found that groups that may be at higher risk for persistent depressive symptoms include individuals with lower educational attainment and current smokers. However, it is also possible that those with depressive symptoms are more likely to smoke. Our findings support the need to evaluate the role of systematic screening for depressive symptoms among patients with CKD.

A major strength of this study was the use of group-based trajectory analysis, which allowed us to characterize participants on the basis of the long-term course of their depressive symptoms. Although it is generally reasonable to use predefined cutoffs (*e.g.*, BDI of 11) to categorize individuals into different groups, this approach has some limitations. First, this approach may create groups that reflect only random variation and cannot test whether the groups construct on the basis of predefined BDI cutoffs are potentially different, a fundamental shortcoming. Second, the uncertainty about an individual's group membership cannot be quantified in the form of probabilities. The group-based trajectory framework overcomes these limitations.^24–26^ One advantage of the group-based trajectory modeling approach is to identify qualitatively distinct patterns that are not readily identifiable using predefined cutoffs. A second closely related advantage, which also stems from the use of a formal statistical structure, is that the methodology has the capacity for distinguishing chance variation across individuals from real differences.

This study has several limitations. First, group membership was estimated based on maximum likelihood, and therefore, not all group members perfectly followed their group trajectory. However, these latent groups are a collection of individuals who follow the same pattern, with only random error producing differences in the trajectories of group members. Therefore, it may be appropriate to consider these group members as similar as possible. Second, the systematic differences between individuals analyzed and those excluded may have introduced selection bias in this study. Third, it remains possible that part of the findings reported in this study may be due to residual confounding. For example, we were unable to adjust for all factors related to social determinants of health; however, we did adjust several individual-level factors, including race and ethnicity, educational attainment, and health insurance. Finally, BDI data were collected at visits that were 2 years apart, and therefore, we were not able to assess variability in depressive symptoms during these time gaps.

In summary, overall, depressive symptoms remained largely stable among individuals with mild-to-moderate CKD and were independently associated with a trajectory of nonlinear, rapid eGFR decline. Our findings suggest that clinicians need to be aware of the presence of persistent depressive symptoms among individuals with CKD and arrange timely treatment and referral to mental health services as needed.

## Supplementary Material

SUPPLEMENTARY MATERIAL

## Supplemental Material

Supplemental Table 1. Characteristics of included and excluded participants.
