# Supplementary material for: Association between Depressive Symptom Trajectory and Chronic Kidney Disease Progression: Findings from the Chronic Renal Insufficiency Cohort Study
Source: Kidney360. 2023 Feb 23;4(5):606–14. doi: 10.34067/KID.0000000000000087 (PMC10278792; doi:10.34067/KID.0000000000000087)
Supplement: SUPPLEMENTARY MATERIAL [file kidney360-4-606-s001.pdf]

Supplemental Table 1: Characteristics of included and excluded participants

Variables	Included participants (n=2361)	Excluded participants (n=1578)
Age, mean (SD), years	58.3 (10.3)	56.7 (11.9)
Sex, Female, %	1109 (47.0)	669 (42.4)
Race, %		
non-Hispanic White	1156 (49.0)	482 (30.5)
non-Hispanic Black	900 (38.1)	750 (47.5)
Hispanic	305 (12.9)	346 (21.9)
Diabetes, %	995 (42.1)	913 (57.9)
Hypertension, %	1975 (83.7)	1416 (89.7)
Cardiovascular disease, %	700 (29.7)	616 (39.0)
BDI Score		
Year=0 (baseline)		
% Responders	2342 (99.2)	1547 (98.0)
Mean (SD)	7.1 (7.1)	9.5 (8.8)
Year=2		
% Responders	2311 (97.9)	751 (47.6)
Mean (SD)	7.0 (7.1)	8.8 (8.3)
Year=4		
% Responders	2295 (97.2)	236 (15.0)
Mean (SD)	7.2 (7.5)	8.6 (9.2)
Year=6		
% Responders	1825 (77.3)	150 (9.5)
Mean (SD)	6.9 (7.0)	8.1 (8.2)
Year=8		
% Responders	948 (40.2)	68 (4.3)
Mean (SD)	6.7 (7.0)	6.8 (6.8)
eGFR, mean (SD), mL/min/1.73 m^2		
Year=0 (baseline)		
% Responders	2361 (100.0)	1564 (99.1)
Mean (SD)	45.6 (14.8)	37.1 (15. 3)
Year=1		
% Responders	2309 (97.8)	1102 (69.8)
Mean (SD)	44.1 (15.2)	33.2 (16.4)
Year=2		
% Responders	2294 (97.2)	735 (46.6)
Mean (SD)	43.4 (15.8)	32.7 (17.5)
Year=3		
% Responders	2245 (95.1)	497 (31.5)
Mean (SD)	42.0 (16.4)	32.2 (18.7)
Year=4		
% Responders	2199 (93.1)	223 (14.1)
Mean (SD)	41.7 (17.8)	38.0 (17.5)
Year=5		
% Responders	2001 (84.8)	192 (12.2)
Mean (SD)	42.7 (18.4)	38.8 (18.1)
Year=6		
% Responders	1833 (77.6)	156 (9.9)

Mean (SD)	43.4 (18.8)	40.1 (18.5)
Year=7		
% Responders	1647 (69.8)	138 (8.8)
Mean (SD)	43.9 (18.9)	40.0 (18.9)
Year=8		
% Responders	1460 (61.8)	128 (8.1)
Mean (SD)	44.3 (18.9)	40.7 (18.7)
